# Applying digital storytelling in the medical oncology curriculum: Effects on students’ achievement and critical thinking

**DOI:** 10.1016/j.amsu.2021.102528

**Published:** 2021-06-29

**Authors:** Afagh Zarei, Rita Mojtahedzadeh, Aeen Mohammadi, John Sandars, Seyed Amir Hossein Emami

**Affiliations:** a Department of Medical Education, Medical School, Tehran University of Medical Sciences, Tehran, Iran; b Tehran University of Medical Sciences, Tehran, Iran; c Edge Hill University, Medical School, Ormskirk, UK; d Department of Internal Medicine, Associate Professor in Hematology/ Medical Oncology Ward, Imam Khomeini Hospital, Tehran University of Medical Sciences, Tehran, Iran

**Keywords:** Digital storytelling, Critical thinking, Medical education

## Abstract

**Background:**

Digital storytelling (DST), which combines traditional storytelling with digital tools, can provide a narrative pedagogy that promotes critical thinking (CT). However, we found no previous study in medical education.

**Materials and methods:**

The aim of the study was to investigate if DST can promote CT and, if so, which CT skills were improved. Thirty-two students participated in a non-equivalent control group pretest-posttest research study, with 16 in each group. The participants were fifth-year medical students on a hematology rotation. We compared the routine instructional method (control group) with DST (intervention group). The measures of CT used for the pre- and post-test in both groups was the Health Science Reasoning Test (HRST) and knowledge test. We also evaluated the satisfaction of the students in DST group. We used Paired and independent t-tests for comparing the mean scores. To eliminate the confounding effect of pre-test on the results of the intervention, the ANCOVA test was used.

**Results:**

There was no significant difference in the overall CT pretest scores (P-value = 0.51) between the control and intervention groupsbut the difference was significant for the post-test scores (P-value = 0.03). Although post-test scores showed a significant increase (P-value = 0.002) compared to pre-test scores in the intervention group, no significant increase was observed in the control group (P-value = 0.26). Most students considered that DST improved their CT, deep learning, communication skills and team-working.

**Conclusions:**

The study demonstrated that DST promoted CT. We recommend the use of DST to promote CT in clinical education placements.

## Highlights

• Critical thinking (CT) is a vital ability for medical students.
• Digital storytelling (DST) is a narrative approach to promote CT.
• DST promote CT as well as analysis skill in clinical setting.
• For medical students who deal with everyday patient's stories, DST is a powerful teaching and learning method in clinical education.

## 1 Introduction

Decision-making is a highly complex skill required for oncology placement. Decisions in this placement are mostly influenced by contextual factors, decision-maker characteristics, and the nature of the decision itself, which may lead to disagreements regarding the best action. Therefore, oncology decisions are based on not only evidence-based medicine, but also clinical experience and the best available research [1]. Meanwhile, a vital skill for medical students in making a diagnosis, deciding on treatments, and avoiding mistakes is critical thinking (CT) [2]. CT is “the ability to apply higher cognitive skills and/or the disposition to be deliberate about thinking that leads to action that is logical and appropriate” [3]. It is closely related to clinical reasoning, problem-solving, and diagnostic reasoning [4], and is an essential skill for making effective judgments [3]. The Delphi panel of the American Philosophical Association (APA) has identified interpretation, analysis, evaluation, inference, explanation, and self-regulation as the core CT skills that should be developed through education [5].

Different pedagogical approaches may be adopted to develop CT. An approach suggested by Cooper (2000) is narrative pedagogy [6] in which teachers and students collaborate to interpret shared experiences to meet educational goals. In this approach, students consider multiple views, question the assumptions and values, and interpret their experiences in different ways [7].

An important narrative pedagogic teaching strategy is digital storytelling (DST) [8] which combines traditional storytelling with text and digital multimedia [9]. According to Morra (2013), educational DST has eight steps: 1) forming an idea, 2) exploring and learning, 3) making a script, 4) storyboarding, 5) compiling images, audios, and videos, 6) putting them together, 7) sharing the digital story, and 8) receiving feedback [10]. Although some studies have concluded that DST can improve some aspects of CT such as reflection [11] and holistic thinking skills [12], there are still some gaps in the current understanding about its potential effectiveness in medical education. The results of a systematic review in health professions education noted few empirical studies on DST, and the main outcome measures were self-reported perceptions of its value [13]. In addition, previously published studies have mainly focused on non-medical higher education [14] or secondary education [15]. Another review showed that only three studies have deployed DST in medical education [16]. These studies were conducted by Sandars (2009), applying DST as a reflection tool for first-year medical students [11]; D'Alessandro (2004) applying DST as a patient simulation; and Codd (2018) considering DST to develop confidence and patient-centered learning [17]. In addition, nowadays, storytelling is recognized as a meaningful approach in cancer care [18].

Considering the above-mentioned gaps in the current understanding, this study aimed to investigate the effectiveness of DST on medical students’ knowledge acquisition and CT skills in oncology placement.

## 2 Methods

This study was a non-equivalent control group pre- and posttest study conducted on two cohorts of fifth-year medical students during a two-week clinical hematology and oncology rotation. In this university, students spend four months on internal medicine, which includes eight distinct rotations, including a two-week hematology and oncology rotation. In the hematology and oncology ward with 27 beds, 10 faculty members are responsible for providing instruction to students and residents in the inpatient and outpatient setting. In this placement, students are expected to become capable of diagnosing and managing the cases mentioned in the curriculum. In addition, it is expected that students acquire CT skills for clinical reasoning. Thirty-two students participated in the study and were assigned to control and intervention groups (n = 16 per group). To prevent contamination in the two groups, first, the control group and then the intervention group were included. The sample size was determined to ensure a significance level of 0.05 and a power of 0.8 [19,20].

### 2.1 Instruments

We used two instruments to identify the effectiveness of DST.

#### 2.1.1 Health sciences reasoning test (HSRT)

To evaluate CT, we used the 33-point form of the HSRT developed by Insight Assessment, a division of California Academic Press. The HSRT is based on the APA Delphi definition of CT [5] and consists of 33 questions that provide an overall measure of CT and its main domains required for reasoning and decision-making, including induction, deduction, analysis, inference and evaluation. The required time to answer the HSRT is 45 min. The valid and reliable original Persian version of HSRT was provided by Insight Assessment. An overall score of 15–20, 21–25, and >26 is indicative of moderate, strong, and superior CT skills, respectively. Subscale scores >5 are considered strong for analysis, inference, and evaluation, and subscale scores >8 are considered strong for induction and deduction [21].

#### 2.1.2 Knowledge test

We developed a 40-item best-answer test, a format of the multiple-choice test, for knowledge assessment. The test covered the intended learning objectives of the hematology and oncology placement and was approved by two hematology and oncology professors other than the research team. The reliability of this tool was calculated using the Kuder-Richardson formula which is a special type of Cronbach's alpha to estimate with dichotomous items [22]. This value for the knowledge test was 0.77 which was acceptable [23].

### 2.2 Instructional design in the control group

On the first day, the participants completed the informed consent form and responded to the HSRT and knowledge test as the pre-test. Then, they participated in the routine teaching-learning process of the ward, which included some lecture-based classes, taking part in clinical rounds, and visiting patients in clinics accompanied by residents and attending physicians. On the last day, they once again took the HSRT and the knowledge test as the post-test.

### 2.3 Instructional design in the intervention group

On the first day, similar to the control group, the participants completed the informed consent form and the HSRT and knowledge test as the pre-test. They were then randomly divided into four groups of four students. A medical case was assigned to each group. These cases included breast cancer, gastric cancer, leukemia, and multiple myeloma. The students received a guideline and samples of digital stories to become familiar with performing DST, including how to create a scenario, a storyboard, a digital story, and evaluate the final product. Moreover, Robin's rubric [24] was discussed with all the groups at the beginning of the rotation. This rubric provides a guideline that evaluates some aspects of a digital story, such as the purpose of the story, the point of view, the dramatic question, the choice of content, the clarity of the voice, the pacing of the narrative, the creation of a meaningful audio soundtrack, the quality of images, the economy of story detail, and the grammar and language usage [24]. Each group explored the case and wrote a scenario including background information, medical history, signs and symptoms, and other related information. Then, the group presented each scenario in the first feedback session under the supervision of its teachers. Based on the feedback received from the teachers and other students, the group revised its story.

Once the scenario was confirmed, a storyboard was created using Microsoft PowerPoint 2019. For this purpose, the group determined appropriate assets, including text, image, sound, or video within the slides needed for each DS. Then, the group started to collect the required assets by taking real photos, self-recording of narratives and videos, or using free materials available on the Internet. In the next step, the students put the assets together to create a digital story in the MP4 format using software such as iMovie or Microsoft PowerPoint 2019.

In the second feedback session, the representative of each group presented the produced digital story. The teachers and other students evaluated the presented digital story with Robin's rubric [24]. The groups revised their digital stories and reshared them based on the received feedback. In this way, the students produced their digital stories within two weeks. On the final day, each student completed the HSRT and knowledge test as the post-test.

### 2.4 Analysis

The Shapiro-Wilk test was conducted to assess the normal distribution of all variables. Measures of the demographic characteristics and dependent variables between the intervention and control groups were investigated with paired and independent t-tests. Analysis of covariance (ANCOVA) was used to compare the mean differences between the groups, in which the pre-test scores of HSRT and the knowledge test were used as the covariate. The level of significance was set at P < 0.05. The data were analyzed using IBM SPSS Statistics for Windows, Version 21.0.

## 3 Results

Thirty-two students participated in this study and completed the pre and post-tests. According to the HSRT manual [21], the scores with the following characteristics (false scores) were omitted from the analysis: (1) test duration time <15 min, (2) an answer rate of <60%, and (3) a three-point decrease from the pre-test to post-test scores. Finally, the scores of 12 students in the control group and nine students in the intervention group were included in the analysis. Table 1 shows the demographic comparison of participants whose scores were included in the analysis.

**Table 1:** Comparison of the participants' sex and age.

Group	N(percent)	Sex		Sig^a^	Age			Sig^b^
		Female	Male		Mean (year)	Std Deviation	Std Error	
Control	12 (57.14%)	7(58.3%)	5(41.7%)	0.52	22.2	0.492	0.142	1.00
Intervention	9 (42.86%)	4(44.4%)	5(55.6%)		22.33	0.5	0.167	

a Chi-square.

b Independent *t*-test.

The Shapiro-Wilk test (P-value >0.05), kurtosis and skewness, and Q-Q plot showed that all variables have an approximately normal distribution. So, we used parametric tests to investigate the differences of means in control and intervention groups. The non-parametric test (Mann-Whitney U) revealed the same results as the independent sample T-test. The pre-test results in the intervention and control groups were compared using an independent *t*-test. The results showed no significant difference between the two groups in terms of HSRT components and knowledge scores (P > 0.05). Then, the pre- and post-test scores in each group were compared using a paired *t*-test (Table 2). Finally, post-test scores were compared with an independent *t*-test, the results of which showed a significant difference between the two groups in terms of analysis (t = −2.58, P = 0.01).

**Table 2:** Comparing the pre- and post-test scores in the two groups.

HSRT component	Control group				Intervention group			
	Mean diff	t	Df	sig	Mean diff	t	df	sig
Induction	0.08 ± 1.24	0.23	11	**0.82**	0.88 ± 1.05	2.53	8	**0.03**
Deduction	−0.33 ± 0.65	−1.77	11	**0.10**	−1.22 ± 1.48	−2.47	8	**0.03**
Analysis	−0.25 ± 0.96	−0.89	11	**0.38**	−1.11 ± 1.05	−3.16	8	**.013**
Inference	0.0 ± 1.34	0.00	11	**1.00**	−0.33 ± 1.00	−1.00	8	**0.34**
Evaluation	−0.16 ± 1.02	-.56	11	**0.58**	0.33 ± 1.11	0.89	8	**0.39**
Overall	−0.58 ± 1.72	−1.16	11	**0.26**	−1.77 ± 1.20	−4.43	8	**0.002**

To eliminate the confounding effect of the pre-test on the results of the intervention, after examining the statistical assumptions, we considered the type of intervention as the independent variable, the post-test results as the dependent variable, and the pre-test results as the covariate. The results of ANCOVA showed that DST had a significant effect on analysis (F = 6.87, P = 0.01, Eta = 0.27) and overall CT (F = 4.99, P = 0.03, Eta = 0.21).

In case of knowledge change in the control and intervention groups, the results are given in Table 3.

**Table 3:** Comparison of knowledge pre- and post-test scores in the two groups.

Variable	Group	Pre-test	Post-test	Sig^a^	ANCOVA			
		Mean ± SD	Mean ± SD		F	P-Value	Eta square	Observed Power
Knowledge	Control	2.5 ± 1.12	10.1 ± 2.44	0.00	F:3.69	0.55	0.02	0.089
	Intervention	2.2 ± 1.42	9.7 ± 3.07	0.00				
Sig^b^		0.55	0.75					

a Paired *t*-test.

b Independent *t*-test.

## 4 Discussion

Clinical reasoning which is the process of patient data collection, prioritizing, developing a hypothesis, and planning to confirm or refuse it depends on CT skills [25]. We investigated the effect of DST on the CT skills of induction, deduction, analysis, evaluation, inference, overall CT, as well as knowledge achievement among medical students. The results revealed that DST enhanced the overall (P = 0.03) and analysis (P = 0.01) scores among other CT components in the intervention group compared to the control group.

A review of the literature indicated that few studies have investigated the impact of DST on CT in medical, nursing, and high-school students [11,12,15,26]. All of these studies reported the development of CT as a result of deploying DST. These findings are aligned with the findings of the present study. Herein, the students in the DST group showed a significant increase in their analysis skills, which was in line with the finding of Tatli's (2017) study on nursing education [27]. Tatli compared the effect of creating a storyboard with paper and pencil instead of digital tools for case analysis on nursing students. The results showed that DST affects the analysis skill. Tatli's study also suggested that digitization can help enhance students' analytical skills. Cooper (2000) also emphasized the role of narratives in better analyzing the topics [6]. Cooper's study supports the power of using narrative in DST to improve the analysis skill. These findings indicate that two features of DST, i.e., digital and narrative features, have the potential to promote the analysis skill and consequently, CT.

Another relevant study was conducted on nursing education by Gazarian (2010) [12] who used DST as an assignment to develop synthesis and CT in a nursing course [12]. Furthermore, the most relevant study to ours in which CT and its components were evaluated was conducted by Yang [15] (2011) who investigated the application of DST on English achievement and CT of high-school students in an English class. Using a critical thinking test (CTT-I) to measure CT, it was shown that the components of interpretation and evaluation of arguments were improved by DST (P < 0.05). Although the CT subscales in CTT-I were different from those in HSRT, the findings of Yang's study were generally aligned with those of the present study.

Although we found a significant increase in the overall CT and analysis skills scores, we did not identify any significant difference between the scores of knowledge and other CT components. Since professors in this study were experienced medical educators with high teaching quality scores and have a tight instructional program in this placement, it is assumed that this is a reason for DST's lack of significant influence on knowledge acquisition compared to the routine teaching method.

The main limitation of the present study was the small sample size and the short duration of the intervention (only two weeks). These limitations may be the other reasons for the lack of a significant difference in some variables between the two groups. In the case of sample size, as mentioned in the Results section, we had to eliminate the false scores in analyzing the data and consequently observed a decrease in the number of included participants. Therefore, further studies with larger samples or with longer durations are recommended. We also suggest qualitative studies to deeply understand the learning process in DST.

This study is noteworthy in that it is the first study in medical education for CT conducted in a real-life context and in comparing the intervention with the routine teaching approach. Another strength is the use of HSRT, which is a specific tool for measuring CT in the health profession setting [21].

## 5 Conclusion

Based on the improvement in overall CT and analysis skills, this study suggests that DST has the potential to develop CT skills. The more affected skill in this study was analysis skill. However, it is not clear whether the lack of a significant change in the scores of other CT skills is due to the lack of impact of DST or other reasons, including the shortness of the rotation as well as the low sample size. In addition, there was no increase in knowledge achievement. There may be several reasons for this, one of which is the impact of the routine educational program in that ward. Therefore, further research is recommended with more students and across different medical placements. The comparison of DST with other teaching and learning methods is suggested as well.

## Ethical approval

This study was performed in line with the principles of the Declaration of Helsinki. Approval was granted by the Ethics Committee of Tehran University of Medical Sciences under the code ID. IR. TUMS.IKHC.REC.1398.238.

## Sources of funding

This study was part of a PhD dissertation in medical education at Tehran University of Medical Sciences with the number 42015 and conducted by its financial support.

## Author contribution

All authors contributed to the study conception and design. Material preparation, data collection and analysis were performed by Afagh Zarei, Rita mojtahedzadeh, Aeen Mohammadi, John Sandars and Seyed Amir Hossin Emami. The first draft of the manuscript was written by Afagh Zarei and Rita Mojtahedzadeh and all authors commented on previous versions of the manuscript. All authors read and approved the final manuscript.

## Consent

Informed consent was obtained from all individual participants (medical students) included in the study.

## Registration of research studies

Not applicable.

## Guarantor

Dr. Seyed Amir Hossein Emami: Department of Internal Medicine, Associate professor in Hematology/Medical Oncology ward, Imam Khomeini Hospital, Tehran University of Medical Sciences, Tehran, Iran. Corresponded author: Tel: 09121063743, emamiami@yahoo.com, Orcid ID; 0000-0002-2253-2185.

## Declaration of competing interest

The authors declare that they have no conflict of interest.
